# Association Between Psychological Distress and Sleep Quality in Children and Adolescents: A Cross-Sectional Study in Zhejiang, China

**DOI:** 10.3390/metabo16040249

**Published:** 2026-04-06

**Authors:** Tian Liang, Zhengmao Zhuang, Lizhi Wu, Xueqing Li, Zhijian Chen, Mingluan Xing

**Affiliations:** 1 Department of Environmental Health, Zhejiang Provincial Center for Disease Control and Prevention, Hangzhou 310051, China; tianliang@mail.ccmu.edu.cn (T.L.); zhengmao.zhuang@mail.ccmu.edu.cn (Z.Z.); lzhwu@cdc.zj.cn (L.W.); xqli@cdc.zj.cn (X.L.); zhjchen@cdc.zj.cn (Z.C.); 2 School of Public Health, Capital Medical University, Beijing 100069, China

**Keywords:** children and adolescents, psychological factors, sleep quality, pediatric females, total triiodothyronine

## Abstract

**Background**: Psychological distress has been increasingly recognized as an important determinant of sleep quality in children and adolescents. However, susceptible subgroups have not been clearly identified. This study aimed to examine the associations between psychological distress and sleep quality in children and adolescents. **Methods**: We conducted a cross-sectional study among 5771 school-aged children and adolescents (6–18 years) in Zhejiang Province. Psychological status was assessed using the Chinese version of the Depression Anxiety Stress Scale-21, and sleep quality was evaluated using the Chinese version of the Pittsburgh Sleep Quality Index (PSQI). Thyroid-related biomarkers were measured via chemiluminescence immunoassay. Associations between psychological distress and sleep quality were analyzed using generalized linear models. In addition, stratified analyses were performed to identify potentially susceptible subgroups by age, sex, and BMI-for-age z score. **Results**: Each one-point increase in depression, anxiety, and stress scores was associated with an increase in PSQI scores of 0.18 (95% CI: 0.16, 0.19), 0.20 (95% CI: 0.18, 0.21), and 0.20 (95% CI: 0.19, 0.22), respectively. Subgroup analyses showed that the associations were more pronounced among older children (age > 12 years) and pediatric females. Exploratory mediation analyses suggested a possible but very limited indirect role of T3 in the associations of depression, anxiety, and stress with sleep quality, with all estimated proportions mediated below 1%. **Conclusions**: Psychological distress was significantly associated with poorer sleep quality in children and adolescents, particularly among older individuals and pediatric females. These findings highlight the importance of early identification and intervention for psychological distress to improve sleep health in younger populations. Further longitudinal studies are needed to clarify underlying mechanisms.

## 1. Introduction

Adequate sleep is fundamental to healthy brain maturation, emotional regulation, and physical growth throughout childhood and adolescence [1]. Despite its importance, sleep disturbances affect an estimated 20–30% of youths worldwide, with substantial consequences if left untreated. Chronic sleep problems not only contribute to impaired neurocognitive functioning and heightened susceptibility to mental health disorders but have also been linked to adverse long-term health outcomes, such as obesity, metabolic syndrome, and cardiovascular disease [1,2].

Mounting evidence suggests a strong interplay between psychological distress and sleep quality in children and adolescents. Sleep disruptions are frequently accompanied by symptoms of depression, anxiety, and behavioral issues, with the relationship being bidirectional in nature [3,4]. For example, studies from North America have revealed that the vast majority of children with anxiety disorders experience at least one form of sleep disturbance [5,6]. Similarly, the co-occurrence of depression and anxiety among adolescents has been associated with compounded risks for unhealthy behaviors, including chronic sleep loss [7]. Nevertheless, evidence on the association between mental health and sleep in children and adolescents remains limited.

Although growing evidence suggests an association between psychological distress and sleep problems, evidence from generally healthy children and adolescents remains limited [8,9]. In particular, it is unclear whether this association differs across subgroups defined by developmental and demographic characteristics [10,11]. Age- and sex-related differences in emotional regulation, stress responsiveness, and sleep patterns may contribute to heterogeneity in vulnerability, while adiposity, as assessed by BMI-for-age z score, may also influence this relationship through its links with both mental health and sleep [12,13,14]. Identifying such subgroup differences is essential for detecting potentially susceptible populations and may contribute to better sleep and mental health outcomes in children and adolescents [14,15].

Therefore, the present study aimed to examine the association between depression, anxiety, and stress and sleep quality in a large sample of school-aged children and adolescents in Zhejiang Province, China, and to identify potentially susceptible subgroups.

## 2. Materials and Methods

### 2.1. Study Design and Participants

This study utilized the baseline population of the Zhejiang Environmental Health Cohort, which enrolled participants from six cities in Zhejiang Province, including Changxing, Kecheng, Dongyang, Tiantai, Luqiao and Jingning, selected to represent geographic and economic diversity. Recruitment occurred between July 2022 and January 2025 using a stratified multistage random sampling strategy. Detailed sampling procedures can be found in previous studies [16,17,18,19].

This study population consisted of school-aged children and adolescents aged 6–18 years in Zhejiang Province. All participants underwent a standardized physical examination and completed an electronic questionnaire survey under the guidance of trained personnel. For younger children, the questionnaires were completed by parents or guardians to ensure accurate comprehension and reporting. The inclusion criteria were as follows (a) local residents aged between 6 and 18 years; (b) no newly diagnosed tumors or undergoing tumor treatment; (c) no diagnosed mental illness or cognitive impairment; (d) no use of iodine-containing medications or contrast agents within the preceding three months; (e) completion of psychological status and sleep quality questionnaires; and (f) completion of hematological tests.

**Figure 1:**
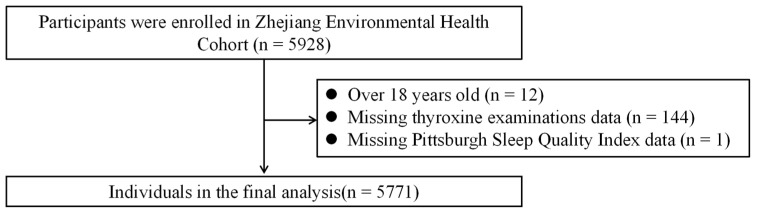
Flowchart of participant selection.

Exclusion criteria included the following: over 18 years old (*n* = 12), missing hematological tests (*n* = 144), and missing Pittsburgh Sleep Quality Index (PSQI) questionnaires (*n* = 1). After exclusions, a total of 5771 participants were included in the final analysis. The flowchart illustrating the inclusion process is shown in Figure 1. The study protocol was approved by the Ethics Committee of Zhejiang Provincial Center for Disease Control and Prevention, and written informed consent was obtained from all participants and their guardians.

### 2.2. Assessment of Psychological Status

Psychological status was assessed using the Chinese version of the Depression Anxiety Stress Scale-21 (DASS-21) [20]. This scale consists of 21 items, divided into three subscales: depression, anxiety, and stress, with 7 items each. Higher scores indicate greater symptom severity in the respective dimensions. The Chinese version of the DASS-21 has been widely validated in Chinese children and adolescents and demonstrates good reliability and validity.

### 2.3. Measurement of Thyroid Hormones

Fasting venous blood samples (5 mL) were collected from each participant in the early morning using serum separation tubes with clot activator. After centrifugation at 3000 rpm for 10 min, serum was separated and stored at −80 °C until analysis. Serum free triiodothyronine (FT3), T3, FT4, total thyroxine (T4), thyroid-stimulating hormone (TSH), triglycerides (Tg), thyroglobulin antibody (TgAb), and thyroid peroxidase antibody (TPOAb) were measured using a chemiluminescence immunoassay. All sample analyses and quality control procedures were performed according to the manufacturer’s instructions.

### 2.4. Outcome Assessment

Sleep quality and disturbances during the previous month were assessed using the PSQI [21]. The PSQI is a 19-item self-rated instrument that has demonstrated good reliability and validity in both clinical and non-clinical settings. These items generate seven component scores: (a) subjective sleep quality; (b) sleep latency; (c) sleep duration; (d) sleep efficiency; (e) sleep disturbances; (f) use of sleeping medication; and (g) daytime dysfunction. The sum of these seven component scores yields a global PSQI score, ranging from 0 to 21. A global score of ≤5 indicates good sleep quality, whereas a score >5 suggests poor sleep quality.

### 2.5. Measurement of Covariates

A standardized and structured questionnaire was used to collect demographic information for each participant, including age and sex. Height, weight, systolic and diastolic blood pressure and heart rate were measured on site at schools by trained research staff. Height was measured using a standard stadiometer, and weight was measured with a medical scale, with participants wearing light clothing and no shoes. Body mass index (BMI, kg/m^2^) was calculated as weight (kg) divided by the square of height (m^2^). BMI-for-age z scores were derived according to the Centers for Disease Control and Prevention growth reference to account for age- and sex-specific growth patterns [22]. Participants were classified as BMI-for-age <85th percentile or ≥85th percentile, with the latter indicating overweight or obesity. Annual household income was categorized as low (<30,000 RMB), middle (30,000–99,999 RMB), high (≥100,000 RMB), or missing, and was included in the adjusted analyses as an indicator of socioeconomic status.

### 2.6. Statistical Analysis

Continuous variables are presented as mean ± SD or median (IQR), and categorical variables are presented as *n* (%). Thyroid-related biomarkers were examined for distributional characteristics before analysis. FT3, T3, FT4, and T4 were approximately normally distributed and were analyzed on their original scale, while TSH, Tg, TgAb, TPOAb, and TRAb showed skewed distributions and were log-transformed before regression and mediation analyses. Normally distributed variables are presented as mean ± standard deviation, whereas skewed variables are presented as median (interquartile range).

To examine the associations between psychological factors (depression, anxiety, and stress) and sleep quality (PSQI global score), generalized linear models were applied. Two models were specified: no covariates were included in crude model, and adjusted model was adjusted for age (>12 vs. ≤12 years), sex (pediatric male/female), BMI-for-age z scores, systolic and diastolic blood pressure and heart rate, in order to account for potential physiological and demographic differences. Results were expressed as linear effect estimates with 95% confidence intervals (CIs) per one-point increase.

In addition, subgroup analyses were performed to investigate the potential modifying effects of age, sex, and BMI-for-age z scores on the relationship between psychological factors and sleep quality. Stratified analyses were conducted according to age (>12 vs. ≤12 years), sex (boy vs. girl), and BMI-for-age z scores (85th percentile vs. <85th percentile). A two-sample Z-test was utilized to assess the homogeneity of the association across various subgroups. For analyses using categorized psychological distress variables, linear trend tests were conducted across the three groups [0 (zero), 1–7 (low non-zero), and >7 (high non-zero)] by modeling the grouped variable as an ordinal variable.

Exploratory mediation analyses were performed to assess the potential indirect role of thyroid-related biomarkers in the associations between psychological distress and sleep quality [23]. Indirect effects were estimated using a bootstrap approach with 1000 resamples. The total effect was decomposed into direct and indirect components based on linear regression models for the exposure–mediator and mediator–outcome associations. A potential mediation effect was considered when the total effect and indirect effect were significant and the proportion mediated was positive.

All analyses were conducted using R (version 4.2.2). All tests were two-sided, and a *p*-value < 0.05 was considered statistically significant.

## 3. Results

### 3.1. Participants’ Characteristics

A total of 5771 participants (mean age 11 ± 2.6 years; 51.7% pediatric males) were included. Most participants were aged 12 years or younger, and the sex distribution was approximately balanced. Detailed characteristics of the study population, including anthropometric measures, vital signs, thyroid-related biomarkers, and psychological and sleep-related scores, are shown in Table 1.

### 3.2. Associations Between Psychological Factors and Sleep Quality

Depression, anxiety, and stress were all associated with poorer sleep quality among children and adolescents aged 6–18 years (all *p* < 0.05). In crude model, higher scores of depression, anxiety, and stress were related to increased PSQI scores, with effect estimates (95% CI) of 0.20 (0.19, 0.22), 0.22 (0.21, 0.24), and 0.23 (0.21, 0.24), respectively. In the adjusted model, each one-point increase in depression, anxiety, and stress was associated with increases of 0.18 (0.16, 0.19), 0.20 (0.18, 0.21), and 0.20 (0.19, 0.22) in PSQI scores, respectively (Table 2).

### 3.3. Subgroup Analysis

Subgroup analyses were conducted to identify subpopulations susceptible to psychological distress. For depression, the association with poorer sleep quality was stronger among children and adolescents older than 12 years than among those aged 12 years or younger (0.29 [0.26, 0.33] vs. 0.11 [0.10, 0.13]). No significant heterogeneity was observed across BMI categories, with similar effect estimates for participants with BMI-for-age ≥85th percentile and <85th percentile. By sex, pediatric males had lower PSQI score increments than pediatric females (0.13 [0.11, 0.15] vs. 0.22 [0.19, 0.24]).

Similar patterns were observed for anxiety and stress. In both analyses, older participants showed stronger associations with poorer sleep quality than those aged 12 years or younger. In addition, pediatric males had lower PSQI score increments than pediatric females. No significant heterogeneity was observed across BMI categories for stress (Table 3).

### 3.4. Mediation Effects of Thyroid Hormones

Exploratory mediation analyses suggested a minimal indirect association involving T3 in the relationships of depression, anxiety, and stress with sleep quality, with proportions mediated all below 1% (Tables S1 and S2).

In the Tables S3 and S4, potential indirect associations involving FT3 and/or T3 were more frequently observed among females, participants aged ≤12 years, and those with BMI-for-age <85th percentile.

### 3.5. Sensitivity Analyses

Sensitivity analyses using three-level categorizations of psychological distress (0, low non-zero, and high non-zero) showed results consistent with the main findings. A clear dose–response relationship was observed, with stronger associations in the high non-zero group compared with the low non-zero group (all *p* for trend < 0.001, Table S5).

## 4. Discussion

In this cross-sectional study of 5764 school-aged children and adolescents (6–18 years) from Zhejiang, China, we found that psychological distress was associated with sleep quality. These associations were consistently observed across depression, anxiety, and stress, and were more pronounced among participants older than 12 years and among pediatric females. The consistency of findings across different categorizations of psychological distress further supports the robustness of the observed associations. Exploratory analyses suggested a minimal indirect association involving T3; however, the magnitude of this potential pathway was small.

In the present study, depression, anxiety, and stress were all associated with poorer sleep quality in children and adolescents. Our findings are consistent with a growing body of literature indicating that sleep disturbances are closely intertwined with emotional problems in pediatric populations. Population-based studies have reported a high prevalence of insomnia symptoms among children and adolescents, with anxiety and depression identified as prominent correlates [24,25,26,27]. Likewise, insufficient sleep has been linked to increased depressive symptoms, while more severe sleep disturbances have been associated with greater internalizing psychopathology and emotional dysregulation [28,29]. Although most of these studies were cross-sectional, the consistency of findings across studies reinforces the close association between psychological distress and sleep problems in young people [30].

In the present study, depression, anxiety, and stress were all significantly associated with poorer sleep quality in children and adolescents. Age- and sex-stratified analyses showed stronger associations in participants older than 12 years and in females, indicating important heterogeneity across pediatric subgroups. Greater vulnerability in participants older than 12 years may reflect developmental changes during adolescence, when academic pressure, peer-related stress, and delayed sleep schedules become increasingly prominent [31]. Consistent with previous studies, pediatric female may be more likely to exhibit internalizing symptoms, including depression and anxiety, and may therefore be more susceptible to the adverse effects of psychological distress on sleep [32,33].

Our findings are consistent with previous evidence showing that depressive, anxious, and stress-related symptoms in children and adolescents are closely associated with sleep problems [31]. In particular, chronic stress, depression, and anxiety have been associated with alterations in both the hypothalamic–pituitary–thyroid axis and the sympathetic nervous system, which may in turn affect metabolic activity, cardiovascular regulation, and central nervous system function [33,34]. Thyroid hormones are involved in multiple physiological processes relevant to sleep regulation, including energy metabolism, autonomic balance, and neuronal signaling [35]. In addition, neuroimaging and translational evidence suggests that sleep patterns are closely related to brain structure and function during adolescence, and that abnormalities in thyroid hormone transport or tissue sensitivity may disrupt sleep-related neural regulation and contribute to anxiety-like behaviors [36].

In our study, exploratory analyses suggested a statistically significant but very small indirect association via T3 in the relationships of depression, anxiety, and stress with sleep quality. Although this finding is biologically plausible in light of the neuroendocrine mechanisms discussed above, the proportion mediated was very low, and therefore caution is warranted when interpreting its generalizability and clinical relevance.

This study has several notable strengths. First, to our knowledge, it is a large-scale population study (*n* = 5771) to systematically investigate the association between psychological distress and sleep quality among children and adolescents. The participants come from different economic regions and geographical areas in Zhejiang Province and have good representativeness. Second, stratified analyses by age, sex, and BMI-for-age z score helped identify potentially susceptible subgroups. Third, the use of extensive covariates adjustment for demographic and physiological factors in the statistical analysis strengthens the robustness of the observed associations and mediation effects.

However, several limitations should be acknowledged. First, the cross-sectional design precluded clarification of the temporal sequence, and the observed associations should not be interpreted causally. Second, although participants were recruited using a stratified multistage random sampling strategy, the findings may not be fully generalizable to children and adolescents in other regions of China. Third, psychological distress and sleep quality were assessed using self- or parent-reported questionnaires, which may be subject to recall bias, and the lack of objective sleep measurements limited the assessment of subtle sleep disturbances. Finally, although several important covariates were adjusted for, residual confounding cannot be excluded, particularly for factors such as pubertal stage, lifestyle behaviors, family environment, and detailed sleep-related characteristics.

## 5. Conclusions

Depression, anxiety, and stress were all found to be significantly associated with poorer sleep quality in children and adolescents, with stronger associations observed in those older than 12 years and in females. These findings suggest that older children and female adolescents may represent particularly vulnerable groups and should receive greater attention in efforts to improve sleep and mental health.

## Figures and Tables

**Table 1:** Characteristics of the study participants.

Demographics	N (%) or Mean ± SD/Median (IQR) (*n* = 5771)
**Age, years**	11 ± 3
>12 years	1588 (27.5%)
≤12 years	4183 (72.5%)
**BMI-for-age z score**	0.03 ± 1.28
**Sex**	
boy	2979 (51.6%)
girl	2792 (48.4%)
**Annual household income**	
Low	261 (4.5%)
Middle	1200 (20.8%)
High	1709 (29.6%)
Missing	2601 (45.1%)
**Vital signs**	
Systolic blood pressure (mmHg)	104.4 ± 12.3
Diastolic blood pressure (mmHg)	65.5 ± 8.1
Heart rate, beats/min (beats/min)	87.5 ± 13.1
**Thyroid hormones**	
FT3 (pmol/L)	6.7 ± 1.3
T3 (nmol/L)	2.5 ± 0.5
FT4 (pmol/L)	17.7 ± 2.8
T4 (nmol/L)	116.4 ± 22.2
TSH (mIU/L)	2.5 (1.8, 3.5)
Tg (ng/mL)	12.1 (8.0, 17.8)
TgAb (IU/mL)	15.6 (13.2, 18.8)
TPOAb (IU/mL)	9.1 (6.7, 11.9)
TRAb (IU/L)	0.4 (0.4, 0.9)
**PSQI score**	1.0 (1.0, 3.0)
**DASS-21 subscales**	
Depression score	0.0 (0.0, 2.0)
Anxiety score	0.0 (0.0, 2.0)
Stress score	0.0 (0.0, 3.0)

*Note*. Continuous variables are presented as mean ± SD or median (IQR), and categorical variables are presented as *n* (%). BMI: body mass index; BMI-for-age z score, age- and sex-standardized body mass index; FT3: free triiodothyronine; T3: total triiodothyronine; FT4: free thyroxine; T4: total thyroxine; TSH: thyroid-stimulating hormone; Tg: thyroglobulin; TgAb: thyroglobulin antibody; TPOAb: thyroid peroxidase antibody; TRAb: thyrotropin receptor antibody; PSQI: Pittsburgh Sleep Quality Index.

**Table 2:** Associations of depression, anxiety, and stress with sleep quality among children and adolescents in Zhejiang, China.

Exposure	Crude Model	*p*	Adjusted Model	*p*
Depression	0.20 (0.19, 0.22)	<0.001	0.18 (0.16, 0.19)	<0.001
Anxiety	0.22 (0.21, 0.24)	<0.001	0.20 (0.18, 0.21)	<0.001
Stress	0.23 (0.21, 0.24)	<0.001	0.20 (0.19, 0.22)	<0.001

*Note*. Crude model: unadjusted. Adjusted model: adjusted for age, sex, BMI, systolic and diastolic blood pressure, heart rate, and annual household income.

**Table 3:** Subgroup analysis for the associations of depression, anxiety, and stress with sleep quality.

Exposure	Subgroup	Effect Estimates, β (95% CI)	*p* for Heterogeneity
Depression	Age		
	>12 year	0.29 (0.26, 0.33)	<0.001
	≤12 years	0.11 (0.10, 0.13)	Reference
	BMI		
	BMI-for-age ≥85th percentile	0.18 (0.15, 0.22)	0.736
	BMI-for-age <85th percentile	0.18 (0.16, 0.20)	Reference
	Sex		
	Pediatric male	0.13 (0.11, 0.15)	<0.001
	Pediatric female	0.22 (0.19, 0.24)	Reference
Anxiety	Age		
	>12 years	0.32 (0.28, 0.36)	<0.001
	≤12 years	0.13 (0.11, 0.15)	Reference
	BMI		
	BMI-for-age ≥85th percentile	0.20 (0.16, 0.23)	0.908
	BMI-for-age <85th percentile	0.20 (0.18, 0.22)	Reference
	Sex		
	Pediatric male	0.14 (0.12, 0.16)	<0.001
	Pediatric female	0.25 (0.22, 0.27)	Reference
Stress	Age		
	>12 years	0.32 (0.29, 0.36)	<0.001
	≤12 years	0.13 (0.11, 0.15)	Reference
	BMI		
	BMI-for-age ≥85th percentile	0.19 (0.16, 0.23)	0.444
	BMI-for-age <85th percentile	0.21 (0.19, 0.23)	Reference
	Sex		
	Pediatric male	0.16 (0.14, 0.18)	<0.001
	Pediatric female	0.24 (0.22, 0.27)	Reference

*Note*. Effect estimates are presented as β (95% CI). Models were adjusted for age, sex, BMI-for-age z score, systolic blood pressure, diastolic blood pressure, heart rate, and annual household income. *p* for heterogeneity indicates the statistical significance of across subgroups.

## Data Availability

The datasets analyzed during the current study are not publicly available due to privacy and confidentiality concerns. However, they are available from the corresponding author upon reasonable request.

## Supplementary Materials

The following supporting information can be downloaded at: https://www.mdpi.com/article/10.3390/metabo16040249/s1, Table S1: Mediation effects of T3 on the associations of depression, anxiety, and stress with sleep quality; Table S2: Mediation analysis of thyroid-related biomarkers in the associations of depression, anxiety, and stress with sleep quality among children and adolescents; Table S3: Stratified mediation analysis showing statistically significant indirect effects in the Zhejiang Environmental Health Cohort; Table S4: Stratified mediation analysis showing non-significant indirect effects in the Zhejiang Environmental Health Cohort; Table S5: Association between psychological distress categorized as zero, low non-zero, and high non-zero and sleep quality among children and adolescents.
