# Supplementary material for: Association Between Psychological Distress and Sleep Quality in Children and Adolescents: A Cross-Sectional Study in Zhejiang, China
Source: Metabolites. 2026 Apr 6;16(4):249. doi: 10.3390/metabo16040249 (PMC13117841; doi:10.3390/metabo16040249)
Supplement: Supplementary file 1 [file metabolites-16-00249-s001.zip › metabolites-4208027-supplementary.pdf]

Supplementary Material

Association Between Psychological Distress and Sleep Quality in Children and Adolescents: A Cross-Sectional Study in Zhejiang, China

Table of Contents

Table S1. Mediation effects of T3 on the associations of depression, anxiety, and stress with sleep quality.

Table S2. Mediation analysis of thyroid-related biomarkers in the associations of depression, anxiety, and stress with sleep quality among children and adolescents.

Table S3. Stratified mediation analysis showing statistically significant indirect effects in the Zhejiang Environmental Health Cohort.

Table S4. Stratified mediation analysis showing non-significant indirect effects in the Zhejiang Environmental Health Cohort.

Table S5. Association between psychological distress categorized as zero, low non-zero, and high non-zero and sleep quality among children and adolescents.

Table S1. Mediation effects of T3 on the associations of depression, anxiety, and stress with sleep quality.

Exposure	Mediator	Average mediation effects	*P*	Total effect	*P*	Proportion	*P*
Depression	T3	0.002 (0.001, 0.003)	0.002	0.177 (0.149, 0.204)	<0.001	0.009 (0.003, 0.018)	0.002
Anxiety	T3	0.002 (0.000, 0.003)	0.006	0.197 (0.170, 0.224)	<0.001	0.008 (0.002, 0.017)	0.006
Stress	T3	0.002 (0.001, 0.003)	0.004	0.204 (0.179, 0.228)	<0.001	0.008 (0.003, 0.015)	0.004

Note. T3: total triiodothyronine. Model adjusted for age, sex, BMI, systolic and diastolic blood pressure, heart rate, and annual household income.

Table S2. Mediation analysis of thyroid-related biomarkers in the associations of depression, anxiety, and stress with sleep quality among children and adolescents.

Exposure	Mediator	Average mediation effects	*P*	Total effect	*P*	Proportion	*P*
Depression	FT3	0.001 (-0.001, 0.002)	0.462	0.177 (0.150, 0.204)	<0.001	0.004 (-0.007, 0.015)	0.462
	FT4	-0.003 (-0.007, -0.001)	0.004	0.177 (0.149, 0.204)	<0.001	-0.019 (-0.039, -0.006)	0.004
	T4	-0.007 (-0.010, -0.004)	<0.001	0.177 (0.149, 0.204)	<0.001	-0.038 (-0.056, -0.025)	<0.001
	TSH	-0.000 (-0.001, 0.001)	0.828	0.177 (0.150, 0.205)	<0.001	-0.000 (-0.004, 0.003)	0.828
	Tg	0.000 (-0.000, 0.001)	0.352	0.177 (0.149, 0.204)	<0.001	0.001 (-0.001, 0.005)	0.352
	TgAb	-0.002 (-0.004, -0.001)	0.002	0.177 (0.149, 0.203)	<0.001	-0.014 (-0.025, -0.005)	0.002
	TPOAb	-0.009 (-0.013, -0.006)	<0.001	0.177 (0.149, 0.203)	<0.001	-0.052 (-0.074, -0.034)	<0.001
	TRAb	-0.003 (-0.005, -0.002)	<0.001	0.177 (0.149, 0.205)	<0.001	-0.019 (-0.031, -0.010)	<0.001
Anxiety	FT3	0.001 (-0.001, 0.002)	0.358	0.197 (0.170, 0.224)	<0.001	0.003 (-0.004, 0.011)	0.358
	FT4	-0.003 (-0.007, -0.001)	<0.001	0.197 (0.170, 0.225)	<0.001	-0.016 (-0.037, -0.005)	<0.001
	T4	-0.007 (-0.009, -0.004)	<0.001	0.197 (0.170, 0.225)	<0.001	-0.033 (-0.048, -0.021)	<0.001
	TSH	-0.000 (-0.001, 0.001)	0.774	0.197 (0.170, 0.225)	<0.001	-0.000 (-0.004, 0.003)	0.774
	Tg	0.000 (-0.000, 0.001)	0.340	0.197 (0.170, 0.225)	<0.001	0.001 (-0.001, 0.004)	0.340
	TgAb	-0.003 (-0.005, -0.001)	<0.001	0.197 (0.169, 0.224)	<0.001	-0.014 (-0.025, -0.006)	<0.001
	TPOAb	-0.009 (-0.013, -0.006)	<0.001	0.197 (0.169, 0.223)	<0.001	-0.046 (-0.066, -0.030)	<0.001
	TRAb	-0.002 (-0.004, -0.001)	0.004	0.197 (0.170, 0.225)	<0.001	-0.011 (-0.020, -0.003)	0.004
Stress	FT3	0.001 (-0.001, 0.002)	0.404	0.204 (0.180, 0.228)	<0.001	0.002 (-0.004, 0.010)	0.404
	FT4	-0.003 (-0.006, -0.001)	<0.001	0.204 (0.179, 0.229)	<0.001	-0.013 (-0.030, -0.004)	<0.001
	T4	-0.006 (-0.009, -0.004)	<0.001	0.204 (0.178, 0.228)	<0.001	-0.030 (-0.044, -0.019)	<0.001
	TSH	-0.000 (-0.001, 0.001)	0.812	0.204 (0.180, 0.229)	<0.001	-0.000 (-0.003, 0.002)	0.812
	Tg	0.000 (-0.000, 0.001)	0.592	0.204 (0.180, 0.228)	<0.001	0.000 (-0.001, 0.003)	0.592
	TgAb	-0.003 (-0.005, -0.001)	<0.001	0.204 (0.179, 0.228)	<0.001	-0.015 (-0.025, -0.007)	<0.001
	TPOAb	-0.008 (-0.011, -0.005)	<0.001	0.204 (0.179, 0.227)	<0.001	-0.038 (-0.056, -0.025)	<0.001

TRAb	-0.002 (-0.004, -0.001)	0.004	0.204 (0.180, 0.229)	<0.001	-0.010 (-0.019, -0.003)	0.004

Notes. FT3: free triiodothyronine; FT4: free thyroxine; T4: total thyroxine; TSH: thyroid-stimulating hormone; Tg: thyroglobulin; TgAb: thyroglobulin antibody; TPOAb: thyroid peroxidase antibody; TRAb: Thyrotropin Receptor Antibody. Models were adjusted for age, sex, BMI-for-age z score, systolic blood pressure, diastolic blood pressure, heart rate, and annual household income.

Table S3. Stratified mediation analysis showing statistically significant indirect effects in the Zhejiang Environmental Health Cohort.

Subgroup	Exposure	Mediator	Average mediation effects	*P*	Total effect	*P*	Proportion	*P*
Sex								
Pediatric female	Depression	FT3	0.002 (0.000, 0.004)	0.016	0.218 (0.172, 0.260)	<0.001	0.009 (0.002, 0.020)	0.016
	Anxiety	FT3	0.002 (0.000, 0.004)	0.014	0.249 (0.203, 0.290)	<0.001	0.008 (0.002, 0.018)	0.014
		T3	0.003 (0.000, 0.005)	0.032	0.249 (0.203, 0.289)	<0.001	0.010 (0.001, 0.021)	0.032
	Stress	FT3	0.002 (0.000, 0.004)	0.014	0.245 (0.203, 0.283)	<0.001	0.008 (0.002, 0.017)	0.014
		T3	0.003 (0.001, 0.005)	0.016	0.245 (0.204, 0.282)	<0.001	0.011 (0.002, 0.022)	0.016
Age								
≤ 12 year	Depression	FT3	0.002 (0.000, 0.003)	0.014	0.116 (0.091, 0.140)	<0.001	0.013 (0.002, 0.026)	0.014
		T3	0.001 (0.000, 0.002)	0.022	0.116 (0.092, 0.139)	<0.001	0.008 (0.001, 0.019)	0.022
	Anxiety	FT3	0.001 (0.000, 0.003)	0.016	0.130 (0.106, 0.154)	<0.001	0.011 (0.002, 0.022)	0.016
		T3	0.001 (0.000, 0.002)	0.028	0.130 (0.106, 0.153)	<0.001	0.007 (0.001, 0.017)	0.028
	Stress	FT3	0.001 (0.000, 0.003)	0.014	0.130 (0.106, 0.153)	<0.001	0.011 (0.002, 0.021)	0.014
		T3	0.001 (0.000, 0.002)	0.038	0.130 (0.106, 0.153)	<0.001	0.006 (0.000, 0.016)	0.038
BMI-for-age								
BMI-for-age <85th percentile	Depression	T3	0.001 (0.000, 0.003)	0.010	0.177 (0.142, 0.210)	<0.001	0.008 (0.002, 0.018)	0.010
	Anxiety	T3	0.001 (0.000, 0.003)	0.028	0.200 (0.165, 0.232)	<0.001	0.007 (0.001, 0.017)	0.028
	Stress	T3	0.001 (0.000, 0.003)	0.020	0.209 (0.178, 0.238)	<0.001	0.006 (0.001, 0.014)	0.020

Notes. FT3: free triiodothyronine; T3: total triiodothyronine. Models were adjusted for age, sex, BMI-for-age z score, systolic blood pressure, diastolic blood pressure, heart rate, and annual household income.

Table S4. Stratified mediation analysis showing non-significant indirect effects in the Zhejiang Environmental Health Cohort.

Subgroup	Exposure	Mediator	Average mediation effects	Total effect	Proportion
Sex					
Pediatric male	Depression	FT3	-0.002 (-0.004, 0.000)	0.130 (0.097, 0.160)	-0.013 (-0.032, 0.000)
		T3	0.000 (-0.001, 0.002)	0.130 (0.096, 0.160)	0.002 (-0.009, 0.017)
		FT4	-0.003 (-0.010, 0.002)	0.130 (0.097, 0.161)	-0.022 (-0.078, 0.019)
		T4	-0.006 (-0.010, -0.003)	0.130 (0.097, 0.160)	-0.047 (-0.081, -0.020)
		TSH	-0.001 (-0.002, 0.001)	0.130 (0.096, 0.161)	-0.005 (-0.019, 0.004)
		Tg	0.000 (-0.001, 0.001)	0.130 (0.096, 0.161)	0.002 (-0.005, 0.010)
		TgAb	-0.003 (-0.006, -0.001)	0.130 (0.097, 0.160)	-0.025 (-0.050, -0.008)
		TPOAb	-0.011 (-0.017, -0.007)	0.130 (0.098, 0.160)	-0.086 (-0.137, -0.049)
		TRAb	-0.003 (-0.005, -0.001)	0.130 (0.097, 0.161)	-0.019 (-0.043, -0.004)
Pediatric female		T3	0.002 (-0.000, 0.005)	0.218 (0.172, 0.259)	0.010 (-0.000, 0.023)
		FT4	-0.003 (-0.006, -0.001)	0.218 (0.172, 0.259)	-0.014 (-0.029, -0.004)
		T4	-0.007 (-0.012, -0.003)	0.218 (0.172, 0.259)	-0.032 (-0.051, -0.016)
		TSH	0.001 (-0.000, 0.002)	0.218 (0.172, 0.259)	0.003 (-0.002, 0.010)
		Tg	0.000 (-0.001, 0.001)	0.218 (0.172, 0.260)	0.000 (-0.003, 0.005)
		TgAb	-0.002 (-0.004, -0.000)	0.218 (0.173, 0.259)	-0.007 (-0.020, -0.000)
		TPOAb	-0.007 (-0.012, -0.003)	0.218 (0.175, 0.258)	-0.031 (-0.054, -0.013)
		TRAb	-0.004 (-0.008, -0.001)	0.217 (0.174, 0.259)	-0.018 (-0.037, -0.005)
Pediatric male	Anxiety	FT3	-0.001 (-0.003, 0.000)	0.140 (0.107, 0.170)	-0.006 (-0.024, 0.003)
		T3	0.000 (-0.001, 0.002)	0.140 (0.107, 0.171)	0.002 (-0.006, 0.015)
		FT4	-0.003 (-0.011, 0.002)	0.140 (0.107, 0.171)	-0.019 (-0.084, 0.013)
		T4	-0.006 (-0.010, -0.002)	0.140 (0.107, 0.171)	-0.040 (-0.071, -0.017)
		TSH	-0.001 (-0.002, 0.001)	0.140 (0.107, 0.171)	-0.004 (-0.018, 0.007)

		Tg	0.000 (-0.001, 0.001)	0.140 (0.107, 0.171)	0.001 (-0.007, 0.009)
		TgAb	-0.004 (-0.007, -0.001)	0.140 (0.107, 0.171)	-0.028 (-0.054, -0.011)
		TPOAb	-0.012 (-0.018, -0.008)	0.140 (0.108, 0.170)	-0.087 (-0.137, -0.051)
		TRAb	-0.002 (-0.005, -0.000)	0.140 (0.107, 0.170)	-0.016 (-0.038, -0.003)
Pediatric female		FT4	-0.003 (-0.006, -0.000)	0.249 (0.204, 0.289)	-0.011 (-0.023, -0.002)
		T4	-0.007 (-0.011, -0.003)	0.249 (0.204, 0.289)	-0.027 (-0.043, -0.013)
		TSH	0.001 (-0.000, 0.002)	0.249 (0.203, 0.290)	0.003 (-0.001, 0.009)
		Tg	0.000 (-0.001, 0.001)	0.248 (0.204, 0.289)	0.000 (-0.004, 0.005)
		TgAb	-0.002 (-0.005, -0.000)	0.248 (0.204, 0.289)	-0.007 (-0.018, -0.001)
		TPOAb	-0.006 (-0.011, -0.002)	0.248 (0.205, 0.289)	-0.024 (-0.043, -0.010)
		TRAb	-0.002 (-0.005, 0.001)	0.248 (0.205, 0.289)	-0.006 (-0.019, 0.004)
Pediatric male	Stress	FT3	-0.001 (-0.003, 0.000)	0.158 (0.129, 0.185)	-0.007 (-0.021, 0.000)
		T3	0.000 (-0.001, 0.002)	0.159 (0.129, 0.185)	0.001 (-0.006, 0.011)
		FT4	-0.003 (-0.009, 0.002)	0.159 (0.129, 0.186)	-0.015 (-0.059, 0.010)
		T4	-0.006 (-0.010, -0.003)	0.159 (0.129, 0.186)	-0.036 (-0.061, -0.016)
		TSH	-0.001 (-0.002, 0.001)	0.159 (0.129, 0.186)	-0.004 (-0.015, 0.005)
		Tg	0.000 (-0.001, 0.001)	0.158 (0.129, 0.186)	0.001 (-0.004, 0.008)
		TgAb	-0.004 (-0.007, -0.001)	0.159 (0.129, 0.186)	-0.023 (-0.044, -0.009)
		TPOAb	-0.010 (-0.015, -0.006)	0.159 (0.129, 0.186)	-0.065 (-0.102, -0.038)
		TRAb	-0.002 (-0.005, -0.000)	0.158 (0.129, 0.186)	-0.013 (-0.029, -0.003)
Pediatric female		FT4	-0.002 (-0.005, -0.000)	0.245 (0.204, 0.282)	-0.009 (-0.020, -0.000)
		T4	-0.006 (-0.010, -0.003)	0.245 (0.204, 0.282)	-0.024 (-0.040, -0.012)
		TSH	0.001 (-0.000, 0.002)	0.245 (0.203, 0.282)	0.002 (-0.002, 0.008)
		Tg	0.000 (-0.001, 0.001)	0.245 (0.203, 0.283)	0.000 (-0.002, 0.003)
		TgAb	-0.002 (-0.005, -0.000)	0.245 (0.204, 0.283)	-0.009 (-0.022, -0.002)
		TPOAb	-0.005 (-0.009, -0.002)	0.245 (0.205, 0.282)	-0.021 (-0.039, -0.008)

		TRAb	-0.002 (-0.005, 0.001)	0.245 (0.204, 0.282)	-0.008 (-0.022, 0.003)
Age					
≤ 12 year	Depression	FT4	-0.003 (-0.005, -0.002)	0.116 (0.092, 0.140)	-0.028 (-0.048, -0.014)
		T4	-0.004 (-0.006, -0.002)	0.116 (0.092, 0.140)	-0.031 (-0.055, -0.013)
		TSH	-0.001 (-0.002, 0.000)	0.116 (0.092, 0.140)	-0.006 (-0.018, 0.003)
		Tg	0.000 (-0.000, 0.001)	0.116 (0.092, 0.140)	0.001 (-0.002, 0.006)
		TgAb	-0.003 (-0.005, -0.002)	0.116 (0.092, 0.139)	-0.029 (-0.047, -0.014)
		TPOAb	-0.011 (-0.015, -0.007)	0.116 (0.092, 0.140)	-0.091 (-0.128, -0.061)
		TRAb	-0.002 (-0.004, -0.001)	0.116 (0.092, 0.141)	-0.016 (-0.032, -0.004)
> 12 year		FT3	-0.000 (-0.006, 0.004)	0.408 (0.351, 0.466)	-0.001 (-0.014, 0.010)
		T3	-0.000 (-0.003, 0.002)	0.408 (0.350, 0.465)	-0.001 (-0.008, 0.004)
		FT4	-0.001 (-0.002, 0.001)	0.408 (0.350, 0.464)	-0.001 (-0.006, 0.002)
		T4	-0.000 (-0.002, 0.001)	0.408 (0.351, 0.465)	-0.000 (-0.005, 0.003)
		TSH	-0.000 (-0.002, 0.001)	0.408 (0.351, 0.465)	-0.000 (-0.004, 0.003)
		Tg	0.000 (-0.001, 0.002)	0.408 (0.350, 0.465)	0.000 (-0.002, 0.005)
		TgAb	0.000 (-0.002, 0.002)	0.408 (0.350, 0.464)	0.000 (-0.005, 0.005)
		TPOAb	0.001 (-0.001, 0.005)	0.408 (0.351, 0.464)	0.003 (-0.002, 0.012)
		TRAb	0.000 (-0.003, 0.004)	0.408 (0.350, 0.464)	0.001 (-0.008, 0.010)
≤ 12 year	Anxiety	FT4	-0.003 (-0.005, -0.001)	0.130 (0.105, 0.154)	-0.023 (-0.039, -0.010)
		T4	-0.004 (-0.006, -0.002)	0.130 (0.105, 0.154)	-0.029 (-0.049, -0.013)
		TSH	-0.001 (-0.002, 0.000)	0.130 (0.106, 0.154)	-0.005 (-0.016, 0.002)
		Tg	0.000 (-0.000, 0.001)	0.130 (0.106, 0.153)	0.001 (-0.001, 0.006)
		TgAb	-0.003 (-0.005, -0.002)	0.130 (0.105, 0.153)	-0.026 (-0.043, -0.013)
		TPOAb	-0.011 (-0.015, -0.007)	0.130 (0.106, 0.154)	-0.082 (-0.114, -0.056)
		TRAb	-0.002 (-0.003, -0.000)	0.130 (0.106, 0.155)	-0.012 (-0.024, -0.003)
> 12 year		FT3	-0.002 (-0.008, 0.002)	0.419 (0.362, 0.471)	-0.003 (-0.020, 0.005)

		T3	-0.001 (-0.004, 0.001)	0.419 (0.362, 0.473)	-0.001 (-0.010, 0.003)
		FT4	-0.000 (-0.003, 0.002)	0.419 (0.362, 0.472)	-0.000 (-0.006, 0.005)
		T4	-0.000 (-0.003, 0.001)	0.419 (0.363, 0.470)	-0.000 (-0.006, 0.003)
		TSH	-0.000 (-0.002, 0.002)	0.419 (0.362, 0.472)	0.000 (-0.004, 0.004)
		Tg	0.000 (-0.001, 0.002)	0.419 (0.361, 0.472)	0.000 (-0.003, 0.005)
		TgAb	-0.000 (-0.003, 0.002)	0.419 (0.362, 0.471)	-0.001 (-0.008, 0.005)
		TPOAb	0.001 (-0.001, 0.005)	0.419 (0.362, 0.471)	0.003 (-0.002, 0.011)
		TRAb	-0.001 (-0.005, 0.004)	0.419 (0.362, 0.471)	-0.002 (-0.013, 0.010)
≤ 12 year	Stress	FT4	-0.003 (-0.005, -0.001)	0.130 (0.106, 0.154)	-0.024 (-0.041, -0.011)
		T4	-0.004 (-0.006, -0.002)	0.130 (0.106, 0.153)	-0.030 (-0.050, -0.014)
		TSH	-0.001 (-0.002, 0.000)	0.130 (0.106, 0.153)	-0.005 (-0.015, 0.002)
		Tg	0.000 (-0.000, 0.001)	0.130 (0.106, 0.152)	0.000 (-0.002, 0.005)
		TgAb	-0.004 (-0.006, -0.002)	0.130 (0.106, 0.153)	-0.028 (-0.044, -0.014)
		TPOAb	-0.010 (-0.014, -0.007)	0.130 (0.105, 0.153)	-0.081 (-0.112, -0.055)
		TRAb	-0.002 (-0.003, -0.000)	0.130 (0.106, 0.155)	-0.011 (-0.023, -0.003)
> 12 year		FT3	-0.001 (-0.006, 0.003)	0.410 (0.362, 0.458)	-0.001 (-0.014, 0.006)
		T3	-0.000 (-0.003, 0.001)	0.410 (0.362, 0.456)	-0.001 (-0.007, 0.003)
		FT4	-0.001 (-0.003, 0.001)	0.410 (0.362, 0.456)	-0.001 (-0.007, 0.003)
		T4	-0.000 (-0.003, 0.001)	0.410 (0.362, 0.455)	-0.001 (-0.006, 0.003)
		TSH	-0.000 (-0.002, 0.001)	0.410 (0.362, 0.457)	-0.000 (-0.004, 0.003)
		Tg	0.000 (-0.002, 0.002)	0.410 (0.362, 0.456)	0.000 (-0.004, 0.005)
		TgAb	-0.001 (-0.004, 0.002)	0.410 (0.362, 0.456)	-0.001 (-0.009, 0.005)
		TPOAb	0.001 (-0.001, 0.004)	0.410 (0.362, 0.455)	0.003 (-0.003, 0.011)
		TRAb	-0.000 (-0.004, 0.004)	0.410 (0.362, 0.455)	-0.001 (-0.009, 0.009)
BMI-for-age group					
BMI-for-age <85th	Depression	FT3	0.001 (-0.001, 0.003)	0.177 (0.142, 0.209)	0.003 (-0.008, 0.016)

percentile		FT4	-0.003 (-0.007, -0.000)	0.177 (0.142, 0.209)	-0.017 (-0.042, -0.002)
		T4	-0.008 (-0.011, -0.005)	0.177 (0.143, 0.209)	-0.043 (-0.062, -0.026)
		TSH	-0.000 (-0.001, 0.001)	0.178 (0.142, 0.208)	-0.000 (-0.005, 0.003)
		Tg	0.000 (-0.000, 0.001)	0.177 (0.142, 0.209)	0.002 (-0.001, 0.007)
		TgAb	-0.002 (-0.005, -0.001)	0.177 (0.143, 0.209)	-0.013 (-0.026, -0.004)
		TPOAb	-0.009 (-0.013, -0.006)	0.177 (0.144, 0.209)	-0.052 (-0.077, -0.030)
		TRAb	-0.004 (-0.006, -0.002)	0.177 (0.143, 0.208)	-0.020 (-0.035, -0.008)
BMI-for-age ≥85th percentile		FT3	0.001 (-0.002, 0.005)	0.185 (0.128, 0.236)	0.005 (-0.012, 0.031)
		T3	0.002 (-0.000, 0.005)	0.185 (0.127, 0.237)	0.010 (-0.001, 0.031)
		FT4	-0.002 (-0.006, 0.000)	0.185 (0.126, 0.239)	-0.010 (-0.033, 0.001)
		T4	-0.003 (-0.009, 0.001)	0.185 (0.125, 0.239)	-0.016 (-0.048, 0.009)
		TSH	0.000 (-0.001, 0.002)	0.185 (0.126, 0.237)	0.000 (-0.009, 0.012)
		Tg	-0.000 (-0.002, 0.002)	0.185 (0.127, 0.237)	-0.000 (-0.012, 0.011)
		TgAb	-0.002 (-0.007, 0.002)	0.185 (0.126, 0.237)	-0.009 (-0.041, 0.012)
		TPOAb	-0.007 (-0.015, -0.001)	0.185 (0.127, 0.237)	-0.036 (-0.084, -0.004)
		TRAb	-0.003 (-0.008, 0.000)	0.185 (0.125, 0.237)	-0.015 (-0.044, 0.001)
BMI-for-age <85th percentile	Anxiety	FT3	0.001 (-0.001, 0.002)	0.200 (0.165, 0.232)	0.002 (-0.004, 0.012)
		FT4	-0.003 (-0.007, -0.000)	0.200 (0.165, 0.233)	-0.014 (-0.037, -0.001)
		T4	-0.007 (-0.010, -0.004)	0.200 (0.166, 0.232)	-0.035 (-0.052, -0.020)
		TSH	-0.000 (-0.001, 0.001)	0.200 (0.165, 0.232)	-0.000 (-0.005, 0.003)
		Tg	0.000 (-0.000, 0.001)	0.200 (0.165, 0.232)	0.002 (-0.001, 0.006)
		TgAb	-0.003 (-0.005, -0.001)	0.200 (0.166, 0.232)	-0.013 (-0.026, -0.005)
		TPOAb	-0.009 (-0.013, -0.005)	0.200 (0.167, 0.231)	-0.045 (-0.066, -0.027)
		TRAb	-0.002 (-0.004, -0.001)	0.200 (0.166, 0.230)	-0.011 (-0.023, -0.003)
BMI-for-age ≥85th percentile		FT3	0.001 (-0.003, 0.005)	0.198 (0.143, 0.248)	0.004 (-0.014, 0.029)
		T3	0.002 (-0.000, 0.006)	0.198 (0.142, 0.248)	0.009 (-0.001, 0.030)

		FT4	-0.002 (-0.007, 0.000)	0.198 (0.141, 0.249)	-0.011 (-0.033, 0.001)
		T4	-0.003 (-0.010, 0.001)	0.198 (0.141, 0.250)	-0.016 (-0.045, 0.006)
		TSH	0.000 (-0.001, 0.002)	0.198 (0.141, 0.247)	0.000 (-0.008, 0.011)
		Tg	-0.000 (-0.003, 0.002)	0.198 (0.142, 0.247)	-0.000 (-0.015, 0.011)
		TgAb	-0.002 (-0.008, 0.002)	0.198 (0.142, 0.248)	-0.011 (-0.045, 0.009)
		TPOAb	-0.008 (-0.016, -0.001)	0.198 (0.143, 0.249)	-0.037 (-0.087, -0.006)
		TRAb	-0.002 (-0.006, 0.000)	0.198 (0.142, 0.248)	-0.009 (-0.032, 0.002)
BMI-for-age <85th percentile	Stress	FT3	0.000 (-0.001, 0.002)	0.209 (0.178, 0.237)	0.002 (-0.004, 0.011)
		FT4	-0.002 (-0.006, -0.000)	0.209 (0.178, 0.238)	-0.010 (-0.028, -0.000)
		T4	-0.006 (-0.010, -0.004)	0.209 (0.178, 0.238)	-0.030 (-0.045, -0.017)
		TSH	-0.000 (-0.001, 0.000)	0.209 (0.178, 0.237)	-0.000 (-0.004, 0.002)
		Tg	0.000 (-0.000, 0.001)	0.209 (0.178, 0.237)	0.001 (-0.001, 0.005)
		TgAb	-0.003 (-0.005, -0.001)	0.209 (0.178, 0.237)	-0.014 (-0.026, -0.005)
		TPOAb	-0.008 (-0.011, -0.004)	0.209 (0.179, 0.238)	-0.036 (-0.054, -0.020)
		TRAb	-0.002 (-0.004, -0.000)	0.209 (0.178, 0.236)	-0.009 (-0.020, -0.001)
BMI-for-age ≥85th percentile		FT3	0.001 (-0.003, 0.005)	0.193 (0.142, 0.240)	0.004 (-0.015, 0.030)
		T3	0.002 (-0.000, 0.006)	0.193 (0.140, 0.240)	0.010 (-0.001, 0.029)
		FT4	-0.003 (-0.007, 0.000)	0.193 (0.140, 0.242)	-0.013 (-0.037, 0.000)
		T4	-0.004 (-0.011, 0.001)	0.193 (0.140, 0.242)	-0.020 (-0.053, 0.006)
		TSH	0.000 (-0.002, 0.002)	0.193 (0.141, 0.240)	0.000 (-0.010, 0.012)
		Tg	-0.000 (-0.003, 0.002)	0.193 (0.142, 0.240)	-0.001 (-0.017, 0.012)
		TgAb	-0.002 (-0.008, 0.002)	0.193 (0.141, 0.240)	-0.009 (-0.042, 0.010)
		TPOAb	-0.007 (-0.014, -0.001)	0.193 (0.141, 0.241)	-0.033 (-0.078, -0.004)
		TRAb	-0.003 (-0.007, 0.000)	0.193 (0.140, 0.240)	-0.013 (-0.040, 0.001)

Note. FT3: free triiodothyronine; T3: total triiodothyronine; FT4: free thyroxine; T4: total thyroxine; TSH: thyroid-stimulating hormone; Tg: thyroglobulin; TgAb: thyroglobulin antibody; TPOAb: thyroid peroxidase antibody; TRAb: thyrotropin receptor antibody. Analyses were stratified by age, sex, and BMI-for-age z score. All models were adjusted for age, sex, BMI-for-age z score, systolic blood pressure, diastolic blood pressure, heart rate, and annual household income, except for the corresponding stratification variable. FT3, T3, FT4, and T4 were analyzed on their original scales, whereas TSH, Tg, TgAb, TPOAb, and TRAb were log-transformed before analysis.

Table S5. Association between psychological distress categorized as zero, low non-zero, and high non-zero and sleep quality among children and adolescents.

Exposure	Comparison	Crude model	*P*	Adjusted model	*P*	*P* for trend
Depression	Low non-zero vs 0	1.24 (1.15, 1.34)	<0.001	1.10 (1.00, 1.20)	<0.001	<0.001
	High non-zero vs 0	6.04 (5.67, 6.41)	<0.001	5.68 (5.31, 6.05)	<0.001	
Anxiety	Low non-zero vs 0	1.26 (1.17, 1.35)	<0.001	1.12 (1.03, 1.21)	<0.001	<0.001
	High non-zero vs 0	6.26 (5.91, 6.61)	<0.001	5.92 (5.57, 6.27)	<0.001	
Stress	Low non-zero vs 0	1.20 (1.11, 1.29)	<0.001	1.08 (0.99, 1.17)	<0.001	<0.001
	High non-zero vs 0	5.64 (5.34, 5.94)	<0.001	5.29 (4.99, 5.59)	<0.001	

Note. The crude model was unadjusted. The adjusted model was adjusted for age, sex, BMI-for-age z score, systolic blood pressure, diastolic blood pressure, heart rate, and annual household income. Psychological distress was categorized into three groups: 0, low non-zero (1-7), and high non-zero (>7), based on the median of non-zero DASS-21 subscale scores. P for trend was calculated by modeling the three-level grouped variable as an ordinal term.
